# Synthesis of Quenchbodies for One-Pot Detection of Stimulant Drug Methamphetamine

**DOI:** 10.3390/mps3020043

**Published:** 2020-06-11

**Authors:** Hee-Jin Jeong, Jinhua Dong, Chang-Hun Yeom, Hiroshi Ueda

**Affiliations:** 1 Department of Biological and Chemical Engineering, Hongik University, 2639 Sejong-ro, Jochiwon-eup, Sejong-si 30016, Korea; heejinjeong@hongik.ac.kr; 2 Department of Chemical System Engineering, Hongik University, 2639 Sejong-ro, Jochiwon-eup, Sejong-si 30016, Korea; ysksun9@gmail.com; 3 Key Laboratory of Biological Medicines in Universities of Shandong Province, Weifang Key Laboratory of Antibody Medicines, School of Bioscience and Technology, Weifang Medical University, Shandong 261053, China; dongjh@wfmc.edu.cn; 4 World Research Hub Initiative, Institute of Innovative Research, Tokyo Institute of Technology, 4259 Nagatsuta-cho, Midori-ku, Yokohama, Kanagawa 226-8503, Japan; 5 Laboratory for Chemistry and Life Science, Institute of Innovative Research, Tokyo Institute of Technology, 4259 Nagatsuta-cho, Midori-ku, Yokohama, Kanagawa 226-8503, Japan

**Keywords:** Quenchbody, antibody, methamphetamine, illicit drug, in-situ immunoassay

## Abstract

The problem of illicit drug use and addiction is an escalating issue worldwide. As such, fast and precise detection methods are needed to help combat the problem. Herein, the synthesis method for an anti-methamphetamine Quenchbody (Q-body), a promising sensor for use in simple and convenient assays, has been described. The fluorescence intensity of the Q-body generated by two-site labeling of *Escherichia coli* produced anti-methamphetamine antigen-binding fragment (Fab) with TAMRA-C2-maleimide dyes increased 5.1-fold over background in the presence of a hydroxyl methamphetamine derivative, 3-[(2S)-2-(methylamino)propyl]phenol. This derivative has the closest structure to methamphetamine of the chemicals available for use in a laboratory. Our results indicate the potential use of this Q-body as a novel sensor for the on-site detection of methamphetamine, in such occasions as drug screening at workplace, suspicious substance identification, and monitoring patients during drug rehabilitation.

## 1. Introduction

The issue of drug abuse is increasing worldwide and has been associated with various social problems. Methamphetamine (MA) is a highly addictive psychostimulant that causes adverse biological effects, such as acute toxic effects on the cardiovascular system, acute renal failure, altered behavioral and cognitive functions, and permanent brain damage [1,2]. Therefore, illicit use of MA is prohibited in many countries. An accurate and rapid detection system for MA is required for clinical diagnostics and criminal forensics, such as drug screening at the workplace and monitoring patients during drug rehabilitation.

One of the current methods for quantitating MA is the Duquenois-Levine colorimetric test, in which an indicator reacts with MA and is compared with standardized color charts visually [3]. Such a colorimetric approach is economic and simple, and can be performed without complicated training. However, the visual interpretation affects the accuracy of results, which is occasionally presumptive and limits the quantification. Immunochromatography, which uses a colloidal gold-labeled antibody or antigen as a detection agent provides a simple analytical procedure for drug screening in biological specimens [4]. However, as the test result is typically interpreted by the observation of a color band on the membrane, the determination of a positive or negative result is also subjective. Another method, thin-layer chromatography (TLC) has been used to detect MA. However, TLC has limited accuracy when used on complex samples due to low resolution of separation; thus, the method is typically used for preliminary identification [3]. Chromatographic methods, including gas chromatography and liquid chromatography, can be used in combination with mass spectrometry to provide high precision results [5,6]. However, these methods involve long sample preparation times, complicated experimental procedures that require a high-level of scientific knowledge and advanced skills and expensive instruments. Enzyme-linked immunosorbent assay (ELISA) has been used as a primary tool for the quantification of MA [7]. However, ELISA involves multiple steps, including several washing and incubating steps, which require one or two days to complete the entire procedure. Thus, a highly accurate assay with easy sample treatment is needed for rapid, onsite MA detection.

**Figure 1:**
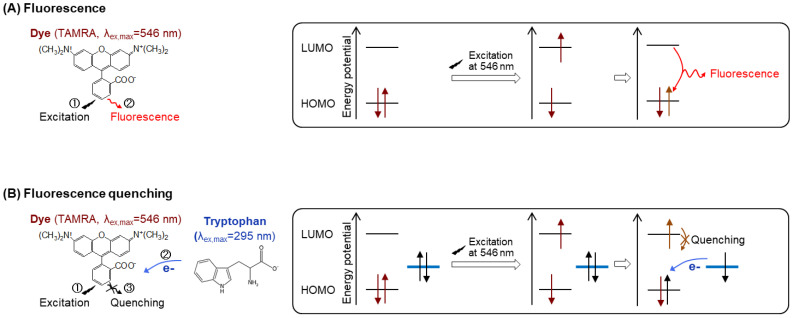
Schematic representation of the mechanisms of (**A**) fluorescence and (**B**) fluorescence quenching.

Antibody-based reagentless fluorescence immunoassays offer the advantages of only taking minutes to complete by eliminating washing steps as well as the use of secondary antibodies. However, these types of assays offer a few challenges in the developmental stage, such as different dye conjugation efficiencies to individual antibodies and non-specific binding to nontarget molecules. For example, labeling an N-hydroxysuccinimide ester-conjugated dye to the lysine (Lys) R-group amines is advantageous due to the numerous Lys residues on the antibody surface. However, this method has the disadvantage such that a good number of Lys residues are distributed all over the antibody, making it difficult to exactly quantify a target antigen due to the random labeling of the fluorescent dye, which is not consistently producing a conjugate product with a known dye:antibody conjugation ratio. Another potential issue is that the Lys residues in the antigen binding region might be conjugated to the dye, which could hamper the antigen-binding activity. To increase the site-specific dye-antibody conjugation efficiency, we developed a new antibody-labeling method using a cysteine (Cys)-containing peptide tag (Cys-tag; MSKQIEVNCSNET [8], a mimic of ProX-tag (MSKQIEVN*SNET, * = amber codon [9]) that was developed for the incorporation of unnatural amino acids), and applied it to the generation of various Quenchbodies (Q-bodies). A Q-body is an innovative immunosensor that fluoresces upon antigen-dependent removal of the fluorophore quenching [9]. In the absence of an antigen, the fluorescence of the dye is quenched by a photo-induced electron transfer from the tryptophan (Trp) residues in the nearby antigen-binding sites of antibody. When an antigen binds to an antibody, the binding stabilizes the conformation of the antibody variable region and the dye associated with the antigen-binding site is sterically hindered from interacting with the Trp, which leads to de-quenching (Figure 1). 

In addition to the dye-Trp interaction, a quenching effect can be achieved by dimerization of two neighboring dyes called H-dimer [10,11,12]. For example, a double-labeled antigen-binding fragment (Fab)-type Q-body, in which the same dye was incorporated in both the H and L chains, showed a higher fluorescent response in the presence of antigen than single-labeled one, which might be due to dye-dye interaction-mediated deeper quenching [8]. The advantage of Q-body-based assays over conventional immunoassays is its simplicity and rapid quantification of various antigens. Q-body assays function in a one-step, one-pot manner. The Q-body reagent is added to the sample and fluorescence is measured after a short incubation time, without any other experimental steps, which is inherent to the conventional immunoassay that needs several incubation and washing steps with blocking agent or additional antibody. Since the fluorescence of the Q-body is quenched in the absence of antigen, its signal-to-background ratio is higher than that of other conventional dye-conjugated antibodies. We have utilized Q-bodies as biosensors for the rapid detection of various targets in a solution, and in some cases on cells, without the need for washing steps [13,14]. Moreover, Q-body assay for detecting BGP peptide was successfully performed not only in the biochemical buffer-based samples but also in 50% plasma with no apparent loss of response [8]. Therefore, Q-body has potential as a universal reagent for monitoring MA in biological fluid such as blood sample. The anti-MA Q-body was previously generated using an amber-codon-based cell-free transcription and translation system. This Q-body showed a dose-dependent response to the MA derivative concentration up to 7.2-fold [8]. However, as the Q-body is produced via an in vitro translation system, the high cost of reagents including unnatural aminoacyl-tRNA-conjugated dye and the low yield remain as obstacles in practical applications.

In this paper, we detail the synthesis of a Q-body using a combination of *Escherichia coli* (*E. coli*) expression for high yields and Cys-tagging for site-specific fluorescence dye conjugation. The conjugating involves the mild reduction of the sulfhydryl group of a Cys-tag followed by labeling with a maleimide-conjugated dye. To develop a one-pot MA detection method, we generated a Q-body that recognizes 3-[(2S)-2-(methylamino)propyl] phenol, a MA derivative with high structural similarity. A major focus was the position and number of the fluorophore, as well as the length of the spacer between the maleimide and dye to synthesize a Q-body with a high response to the antigen. The results indicate the potential use of high yield *E. coli*-based recombinant antibody production and thiol-based antibody labeling with fluorescent dyes against various biomarkers, including MA.

## 2. Experimental Design

The protocol detailed in this paper can be divided to two parts; the expression of anti-MA Fab and its conversion to Q-body. The pUQ1H(MeM9), pUQ1L(MeM9), and pUQ2(MeM9) plasmids are generated and transformed in the *E. coli*, respectively. The expression of anti-MA Fab is induced in a large quantity of bacterial culture. The expressed Fabs are present as soluble form, and are purified using a His-tag at the C-terminus of H chain by immobilized metal affinity chromatography (IMAC). The second part is the labeling of Fab and confirming the efficiency of the Q-body as a probe for detecting MA derivative. After reduction of cysteine residue on the Cys-tag(s) using TCEP agarose beads, its labeling with either TAMRA-C0-maleimide, TAMRA-C2-maleimide, or TAMRA-C5-maleimide is performed via maleimide-thiol reaction. Q-body is purified using His-tag-based IMAC followed by Flag-tag-based affinity purification. The buffer is exchanged to phosphate-buffered saline added with 0.05% Tween20 (PBST) by using 3 k MWCO ultra-filtration column. The fluorescence intensity of Q-bodies with denaturant is measured using a spectrofluorometer to compare their quenching capacities and to select the most efficient type of Q-body through a total of nine variation (three Fab constructs with different numbers and positions of Cys-tag, and three dyes with different length of linkers between TAMRA and maleimide). Next, various concentrations of 3-[(2S)-2-(methylamino)propyl]phenol, phenethylamine, methoxyphenamine, or PBST are added for titration, and fluorescence titration curves are drawn at the emission maxima of each spectrum.

### 2.1. Materials

- KOD-Plus-Neo DNA polymerase (Toyobo, Osaka, Japan; Cat. No.: TOKOD-401)
- Midori Green advance nucleic acid staining solution (Nippon Genetics, Tokyo, Japan; Cat. No.: MG04)
- In-Fusion HD cloning kit (Toyobo, Osaka, Japan; Cat. No.: 639650)
- Restriction enzymes (*Nde*I-HF, and *Bam*HI-HF) (NEB, Tokyo, Japan; Cat. Nos.: R3131, R3136)
- Bacto Tryptone (BD Difco, NJ, USA; Cat. No.: 211705)
- Bacto Yeast Extract (BD Difco, NJ, USA; Cat. No.: 212750)
- Sodium Chloride (Wako, Osaka, Japan; Cat. No.: 191-01665)
- LB Agar (BD Difco, NJ, USA; Cat. No.: 214010)
- Ampicillin sodium (Wako, Osaka, Japan; Cat. No.: 014-23302)
- Sodium phosphate dibasic anhydrous (Wako, Osaka, Japan; Cat. No.: 194-02875)
- Sodium phosphate monobasic dihydrate (Wako, Osaka, Japan; Cat. No.: 199-02825)
- Imidazole (Wako, Osaka, Japan; Cat. No.: 095-00015)
- Potassium chloride (Wako, Osaka, Japan; Cat. No.: 160-03555)
- Plasmid Miniprep kit (Promega, Tokyo, Japan; Cat. No.: A1223)
- SHuffle T7 Express LysY Competent Cells (New England Biolabs, Tokyo, Japan; Cat. No.: C3030)
- Isopropyl β-D-1-thiogalactopyranoside (Wako, Osaka, Japan; Cat. No.: 090-05146)
- Centrifugal filter tube Ultra-4, MWCO 3 k (Millipore, Tokyo, Japan; Cat. No.: UFC800396)
- Immobilized Tris(2-carboxyethyl)-phosphine (TCEP) disulfide-reducing gel (Pierce Biotechnology, Thermo Fisher Scientific, Rockford, IL, USA; Cat. No.: 77712)
- TAMRA-C0-maleimide (AnaSpec, Fremont, CA, USA; Cat. No..: AS-81445)
- TAMRA-C2-maleimide (AnaSpec, Fremont, CA, USA; Cat. No.: AS-81441-5)
- TAMRA-C5-maleimide (Biotium, Hayward, CA, USA; Cat. No.: 91040)
- Ni Sepharose High Performance IMAC resin (GE Healthcare, Piscataway, NJ, USA; Cat. No.: 17526801)
- Empty gravity flow column (Bio-Rad, Hercules, CA, USA; Cat. No.: 7321010)
- Empty spin columns (Bio-Rad, Hercules, CA, USA; Cat. No.: 7326204)
- Anti DYKDDDDK-tag antibody beads (Wako, Osaka, Japan; Cat. No.: 012-22781)
- DYKDDDDK peptide (Wako, Osaka, Japan; Cat. No.: 040-30953)
- Bovine serum albumins (Sigma-Aldrich, Tokyo, Japan; Cat. No.: A2153)
- Unstained protein standards (Bio-Rad, Hercules, CA, USA; Cat. No.: 1610396)
- Dual color protein standards (Bio-Rad, Hercules, CA, USA; Cat. No.: 1610374)
- 3-[(2S)-2-(methylamino)propyl]phenol (NetChem, New Brunswick, NJ).
- Phenethylamine (Sigma-Aldrich, Tokyo, Japan; Cat. No.: 128945)
- Methoxyphenamine (Sigma-Aldrich, Tokyo, Japan; Cat. No.: M1641)

### 2.2. Equipment

- Gel electrophoresis (GELmieru) (Wako, Osaka, Japan; Cat. No.: 290-33891)
- Thermal cyclers for PCR (T100 Thermal Cycler) (Bio-Rad, Hercules, CA, USA; Cat. No.: 1861096)
- Nanodrop (Optizen NanoQ Lite) (KLab, Daejeon, Korea; Cat. No.: S23-3269-031)
- Shaking incubator (Shaking Incubator) (Haneul Techpia; Seoul, Korea; Cat. No.: SI-600R)
- Sonicator (VC750 Ultrasonic Processor) (Sonics&Materials, Newtown, CT, USA; Cat. No.: CV-750)
- Centrifuge with a swinging-bucket rotor (Centrifuge: Combi 514R, Swing rotor: S750T-4B) (Hanil Science, Daejeon, Korea; Cat. No: Combi-514R)
- UV-visible spectrophotometer (Vis Spectrophotometer) (Human Corporation, Seoul, Korea; Cat. No.: X-MA1200V)
- Water bath (High precision Water-Bath) (Changshin Science, Seoul, Korea; Cat. No.: C-AWBP)
- Fluorescence spectrophotometer (FP-8500) (JASCO, Tokyo, Japan; Cat. No.: FP-8500DS)

## 3. Procedure

### 3.1. DNA Cloning (Time for Completion: 14 Days)

To construct a DNA for generating a heavy (H) chain-labeled Fab-type Q-body (H1L0), the pUQ1H vector on which a Cys-tag (MSKQIEVNCSNETG) is encoded at the N-terminus of the H chain of anti-MA Fab. To construct a DNA sequence for generating a light (L)-chain labeled Fab-type Q-body (H0L1), the pUQ1L vector on which a Cys-tag is encoded at the N-terminus of the L chain of Fab. In parallel, to construct a DNA for generating H and L chains-labeled Fab-type Q-body (H1L1), the pUQ2 vector on which two Cys-tags are encoded at the N-terminus of H and L chains of Fab.

#### 3.1.1. Construction of H1L0-Type Anti-MA Q-body Expression Gene

1. Prepare 10 µM diluted DNA primer solutions by mixing 10 µL of 100 µM primer stock with 90 µL ultrapure water. The primer sequences for inserting the anti-MA Fab DNA sequences with the N-terminal Cys-tag into a pUQ1H is Fw-primer for the H chain of Fab (Fd): ProxNdeBack (AAGGAGATATACAtATGTCTAAACAAATCGAAG), Rv-primer for Fd: CH1ForOverlap (ATATCTCCTTCTAGATTATTACTTGTCATCGTCG), Fw-primer for Lch: OverlapProxBack (TCTAGAAGGAGATATCACATGTCTAAACAAATCGAAG), and Rv-primer for Lch: CLforBamHI (CTTGTAGTCGGATCCTTATTAATGATGATGATGATGATGAG)
2. Prepare reaction mix in ice as follows for amplifying H chain of MeM9: 5 µL of dNTPs, 1 µL of 10 µM Fw-primer for Fd, 1 µL of 10 µM Rv-primer for Fd, 50 ng DNA template (tgcHchain) (Table 1), 3 µL of MgSO_4_, 5 µL of Enzyme buffer, 1 µL of KOD-Plus Neo enzyme, and ultrapure water up to 50 µL.
3. Prepare reaction mix in ice as follows for amplifying L chain of MeM9: 5 µL of dNTPs, 1 µL of 10 µM ProxNdeBack, 1 µL of 10 µM CLforBamHI, 50 ng DNA template (tttLchain) (Table 1) 3 µL of MgSO_4_, 5 µL of Enzyme buffer, 1 µL of KOD-Plus Neo enzyme, and ultrapure water up to 50 µL.
4. Combine each mixture in ice in 0.2 mL PCR tube, mix gently and centrifuge briefly (1 s, RT).
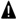
**CRITICAL STEP** It is important to use DNA polymerase with proofreading activity to minimize DNA amplification error.
5. Use the following conditions to amplify the DNA fragments: 94 °C for 2 min, [98 °C for 10 s, Tm for 30 s, 68 °C for 30 s] × 35, hold at 25 °C.
6. Prepare a 1.5% (wt/vol) agarose gel in TAE buffer (40 mM Tris, 20 mM acetic acid and 1 mM EDTA) with Midori Green DNA Gel Stain diluted 1:20,000 (5 µL of dye in 100 mL of TAE).
7. Mix 3 µL of the PCR product with 0.6 µL of 5× gel loading dye and separate by running the gel at 15 V/cm for 30 min. Load 3 µL of 1 kb Plus DNA ladder on the same gel. Then, visualize the DNA fragments using a gel imaging system. The expected PCR product size for H chain is 800 bp and for L chain is 777 bp.
8. Use a NucleoSpin PCR Clean-Up kit according to the manufacturer’s instructions to purify the PCR products.
9. Measure the DNA concentration using UV absorbance on a spectrophotometer (Nanodrop)
10. Prepare reaction mix in ice as follows for combine H chain and L chain: 5 µL of dNTPs, 1 µL of 10 µM Fw-primer for Fd, 1 µL of 10 µM Rv-primer for Lch, 50 ng purified H chain 50 ng purified L chain, 3 µL of MgSO_4_, 5 µL of Enzyme buffer, 1 µL of KOD-Plus Neo enzyme, and ultrapure water up to 50 µL.
11. To ligate the combined Fab fragment into the pUQ1H vector, digest the pUQ0H1L(DON)[15] with *Nde*I and *Bam*HI restriction enzymes using the following mixture: 1 µg DNA, 5 µL NEB CutSmart Buffer (10×), 1 µL of *Nde*I-HF (20 U), 1 µL of *Bam*HI-HF (20 U), and ultrapure water up to 50 µL. After that, incubate the digestion mixture at 37 °C for 1 h. Cool the mixture to 4 °C.
12. Separate the digested pUQ1H DNA vector using a 0.7% (wt/vol) agarose gel at 15 V/cm for 30 min. The expected DNA size for pUQ1H vector is 5404 bp.
13. Extract the desired band and purify it using a NucleoSpin Gel Clean-Up Kit according to the manufacturer’s instructions.
14. Measure the DNA concentration using UV absorbance.
15. Clone the Fab DNA into the pUQ1H vector by setting up the following ligation reaction: 2 µL of Infusion enzyme, 10 ng of insert DNA (Fab), and 10 ng of vector DNA (pUQ1H).
16. Gently mix the reactions and incubate at 50 °C for 150 min. Then, chill the mixture on ice.
17. Transfect the ligation reaction into DH5a chemically competent cells by first thawing the cells (100 µL per vial for each transfection) on ice. Add 10 µL of the ligated mixture from Step 14 to the cells and incubate the mixture for 30 min on ice. After that, incubate the cells for 45 s at 42 °C, and then immediately chill the cells on ice. Add 500 µL of LB medium (10 g Tryptone, 5 g Yeast Extract, 10 g NaCl and ultrapure water up to 1 L) to the cells and incubate them for 1 h at 37 °C with vigorous shaking (200 rpm). Spin it at 10,000 g for 10 min at room temperature in a microcentrifuge and remove 400 µL of the supernatant. After pipetting, spread the cells on pre-warmed LB plate (10 g Tryptone, 5 g Yeast Extract, 10 g NaCl, 15 g Agar and ultrapure water up to 1L) containing 100 µg/mL ampicillin, and then, incubate the plate overnight at 37 °C. Inspect the LB plate for colony formation. Normally, tens of colonies of Fab DNA inserted into pUQ1H is expected on a cloning plate.
18. Use a sterile pipette tip to inoculate five single colonies into five tubes each containing 4 mL of LB medium supplemented with 100 µg/mL ampicillin. Incubate the culture tubes at 37 °C with vigorous shaking (200 rpm) overnight.
19. Use a plasmid DNA purification kit to isolate plasmid DNA from the 4 mL of bacterial culture.
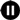
**PAUSE STEP** Plasmid DNA can be stored for several years at −20 °C.
20. Confirm the presence of Fab DNA by Sanger sequencing using T7 promoter-Fw (TAATACGACTCACTATAGGG) and T7 terminater-Rv (GCTAGTTATTGCTCAGCGG) primers.

#### 3.1.2. Construction of H0L1-Type Anti-MA Q-body Expression Gene

To insert a Cys-tag into the N terminus of L chain of anti-MA Fab, H chain was amplified with the primers ProxNdeBack and CH1ForOverlap and tttHchain (Table 1) as a template DNA, and L chain was amplified with the primers OverlapProxBack and CLforBamHI and tgcLchain (Table 1) as a template DNA. The amplified DNA fragments were linked by splice-overlap-extension (SOE) PCR with ProxNdeBack and CLforBamHI, then cloned into the *Nde*I-/*Bam*HI-digested pUQ0H1L(DON) [15]. Follow Steps 1–20.

#### 3.1.3. Construction of H1L1-Type Anti-MA Q-body Expression Gene

To insert Cys-tags into the N terminus of both H and L chains of anti-MA Fab, H chain was amplified with the primers ProxNdeBack and CH1ForOverlap and tgcHchain (Table 1) as a template DNA, and L chain was amplified with the primers OverlapProxBack and CLforBamHI and tgcLchain (Table 1) as a template DNA. The amplified DNA fragments were linked by splice-overlap-extension (SOE) PCR with ProxNdeBack and CLforBamHI, then cloned into the *Nde*I-/*Bam*HI-digested pUQ0H1L(DON) [15]. Follow Steps 1–20.

### 3.2. Fab Expression in E. coli (Time for Completion: 4 Days)


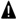
**CRITICAL STEP** All steps in this section must be carried out in a sterile environment.

1. Transform pUQ1H, pUQ1L, or PUQ2 plasmid vector containing anti-MeM9 Fab, Cys-tag(s) at the N-terminal of H and/or L chain, His-tag at the C-terminal of H chain, and Flag-tag at the C-terminal of L chain into SHuffle T7 LysY competent cells. Add 5 µL of plasmid to 100 µL of competent cells and incubate the mixture for 30 min on ice. Incubate the cells for 45 s at 42 °C, and then immediately chill the cells on ice. Add 500 µL of LB medium to the cells and incubate them for 1 h at 37 °C with vigorous shaking (200 rpm). Spin it at 10,000 g for 10 min at room temperature in a microcentrifuge and remove 1 mL of the supernatant. After pipetting, spread the cells on pre-warmed LB plate containing 100 µg/mL ampicillin, and then incubate the plate for 16 h at 37 °C.
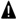
**CRITICAL STEP** Do not overgrow the colonies. This can result in local breakdown of the ampicillin, and formation and survival of satellite colonies that lack the plasmid.
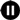
**PAUSE STEP** The plates can be removed from the incubator, sealed in Parafilm, and stored upside down at 4 °C for up to 2 weeks.
2. Add 4 µL of 100 mg/mL ampicillin stock to 4 mL culture tube containing 4 mL of LB medium. Pick a single colony and inoculate it in the culture tube.
3. Incubate the culture tube in a tabletop shaker at 200 rpm at 37 °C. Monitor growth using a UV-visible light spectrophotometer measuring absorbance at 600 nm. Grow the colonies until the optical density (OD) 600 value reaches 0.9 (typically 16–18 h).
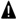
**CRITICAL STEP** Growth rates will be dependent upon the protein of interest. For each protein, it is recommended to experimentally determine the ideal expression time for optimal expression. Overgrowth will deplete the ampicillin, resulting in the growth of cells lacking the plasmid.
4. Add 200 µL of ampicillin stock to 1 L flasks containing either 200 mL of LB medium.
5. Inoculate the medium with 2 mL of confluent 4 mL culture from Step 4 (1 mL per 100 mL of culture). Incubate the culture flask in a shaker at 200 rpm at 37 °C.
6. Monitor growth using a UV-visible light spectrophotometer measuring absorbance at 600 nm. Grow the colonies until the OD600 value reaches 0.6. **Troubleshooting:** If the OD600 value is higher than 0.6, dilute the sample until the value reaches 0.3 and re-incubate the sample until its OD600 value reaches 0.6.
7. Induce the cells with 80 µL of 1 M IPTG in ultrapure water (0.4 mM final concentration) and incubate the culture flasks in a shaker for 16 h at 200 rpm at 16 °C.
8. Transfer the sample to 50 mL centrifuge tubes and pellet the cells by spinning down them at 4000 rpm for 30 min at 4 °C in a centrifuge. Decant the supernatant without disturbing the pellet.
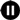
**PAUSE STEP** The pellets can be stored at 4 °C for at least 1 week or −20 °C for at least 4 weeks.

### 3.3. Antibody Purification (Time for Completion: 1 Day)

1. Take the cell pellets from the refrigerator or freezer and allow them to thaw on ice. Add 10 mL of His-binding buffer [50.8 mM Disodium hydrogen phosphate, 18.5 mM Sodium dihydrogen phosphate, 300 mM Sodium chloride (NaCl), pH 7.4] per original 100 mL culture. Mix gently by swirling the buffer by using pipet aid.
2. Lyse the cells by sonicating the pellet on ice with the tapered probe for 10 min of operation time with a 50% duty cycle. Place the end of the tip at about the point where the tube tapers. Adjust the probe setting so that the probe has a low growl sound, rather than a high-pitched whine.
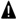
**CRITICAL STEP** It is important to maintain a low temperature (4 °C) during sonication. Therefore, the samples must be sonicated on ice with pulse intervals short enough to prevent overheating the sample when lysing the cells.
3. Transfer the sample to a 50 mL centrifuge tube and spin it at 4000 rpm for 30 min at 4 °C in a centrifuge to pellet the cellular debris.
4. Prepare the His-binding column by packing 200 µL of His-beads into empty gravity column followed by applying 5 column volume (CV) of phosphate-buffered saline (PBS, 145 mM NaCl, 15.5 mM Disodium hydrogen phosphate, 1.7 mM Sodium dihydrogen phosphate, pH 7.4).
5. Apply the supernatant from Step 3 to the column, close the cap of the column, and allow it bind to the beads for 2 h at 25 °C by gently stirring it with a rotator. **Note:** His-tagged Fab and H chain should be retained on the column.
6. Pass through the column by gravity flow and wash the beads by applying 5 CV of His-washing buffer [50.8 mM Disodium hydrogen phosphate, 18.5 mM Sodium dihydrogen phosphate, 300 mM NaCl, 5 mM Imidazole, pH 7.4) to the column. Drain the buffer. Repeat this step three times.
7. Stop the flow and close the cap of column. Apply 1.5 CV of His-elution buffer [50.8 mM Disodium hydrogen phosphate, 18.5 mM Sodium dihydrogen phosphate, 300 mM NaCl, 500 mM Imidazole, pH 7.4). Agitate the resin by gently stirring it for 1 h at 25 °C. Start the flow and collect the elution.
8. Confirm that Fab is in the elution by running an SDS-PAGE and staining with Coomassie blue. (SDS-PAGE gel: 12% Separating gel; 2 mL 30% Acrylamide, 1.25 mL 1.5 M Tris (pH 8.8), 1.65 mL ultrapure water, 25 µL 10% SDS, 50 µL 10% APS, 2 µL TEMED. Staking gel; 165 µL 30% Acrylamide, 125 µL 0.5 M Tris (pH 6.8), 0.7 mL ultrapure water, 10 µL 10% SDS, 10 µL 10% APS, 2 µL TEMED)
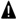
**CRITICAL STEP** We recommend running the eluted sample and the flow-through sample from Step 6 on the same gel for comparison. In the case that the protein of Fab and/or H chain is found in the flow-through sample and not in the elution, re-initialize the His-binding column (Step 4). Repeat Steps 5–7, starting with reapplication of the flow-through that contains the remaining Fab and/or H chain.
9. Exchange the buffer of eluted sample to PBS using a 3 k MWCO Amicon centrifugal ultrafilter. Add the eluted sample in the top part of the filter and centrifuge at 4000 rpm until the volume of the sample is 0.2 mL.
10. Loading 5 mL of PBS and centrifuge at 4000 rpm until 0.2 mL of the sample remains. Repeat this step three times.
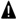
**CRITICAL STEP** Do not let the volume reduce below 0.2 mL. This can cause aggregation of the protein.
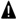
**CRITICAL STEP** Do not let the filters dry out. This may result in poor recovery.
11. Recover the buffer exchanged protein from the membrane, use a 200 µL pipette tip, and insert the tip in the bottom of the filter unit.
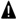
**CRITICAL STEP** Withdraw the sample in several aliquots; be careful not to puncture the membrane.
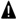
**CRITICAL STEP** Recover the buffer exchanged sample from the filter immediately after final centrifugation to maximize recovery yield.
12. Check the protein expression and protein purity with 10 µL of sample by running an SDS-PAGE. Determine the protein concentration by generating a standard curve of bovine serum albumin (BSA) [16]. Prepare serial dilution, ranging from 0.31 to 1 mg/mL, from a stock solution of 10 mg/mL BSA. Load the purified protein of unknown concentration, molecular mass markers, and multiple BSA samples to each well of gel. Following sample preparation and separation by electrophoresis, the proteins are stained with Coomassie blue. The gel is de-stained to eliminate background, dried between two sheets of cellophane, and scanned with a common desktop scanner, and the resulting image is imported into the freeware program ImageJ (NIH) for analytical analysis. **Troubleshooting:** If you find protein degradation, try to perform the purification steps at 4 °C and/or add protease inhibitors to the cell lysis buffer and purification buffer to prevent degradation.
13. Dilute the dialyzed protein to 1 mg/mL using PBS with 15% glycerol. Prepare aliquots of the samples, freeze them on dry ice, and lyophilize. Store the samples at −80 °C.
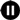
**PAUSE STEP** Lyophilized protein has been stored for several years at −80 °C with no observable effects on protein function or structure upon re-solubilization.

### 3.4. Labeling Fab with Maleimide-Conjugated Fluorescent Dye (Time for Completion: 1 Day)

1. Add 100 µL of TCEP reducing gel slurry to a spin-cup column placed in a microcentrifuge tube.
  Centrifuge at 1000 rpm in a microcentrifuge for 30 s. Remove and discard the supernatant.
2. Apply 100 µL of purified protein to the top of the gel in the tube. Gently vortex or mix the sample and gel, and incubate sample by placing the tube on a rotating wheel to keep the gel in suspension for 1 h (Sample concentration: incubation time, <0.1 mg/mL: 15 min; 0.1–0.5 mg/mL: 30 min; 0.5–0.9 mg/mL: 45 min; >1 mg/mL: 1 h). **Note:** The rotating speed must be sufficient to maintain the gels in suspension. Adjust the rotating speed to not settle the gels and to perfectly mix the gels to proteins.
3. Place an empty spin column in a new tube. Apply the sample with gel, and centrifuge at 1000 rpm for 1 min. **Note:** The collected flow-through is the reduced protein.
4. Prepare a 10 mM TAMRA-maleimide dye stock solution using 10% DMSO.
5. Add a volume of dye stock solution to result in a molar ratio of 20 dyes per Fab. Incubate the reaction solution at RT for 2 h or at 4 °C for 16 h.
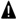
**CRITICAL STEP** the sample needs protection from light.

### 3.5. Purification of Q-body (Time for Completion: 1–2 Days)

1. **Note:** Use anti-Flag tag (DYKDDDDK) antibody immobilization beads to purify DYKDDDDK tagged Q-bodies by the competitive elution of DYKDDDDK peptide. Prepare 20 µL (beads net volume 10 µL) of bead slurry to a tube and centrifuge at 5000× *g* for 1 min.
2. After removing the supernatant, prewash the beads three times by using 500 µL of PBS.
3. Add fluorescent dye-labeled Fab sample into a tube of prewashed beads and mix by rotator at 4 °C for 16 h.
4. Centrifuge the beads at 5000× *g* for 1 min and remove the supernatant.
5. Add 500 µL of PBS, centrifuge the beads at 5000× *g* for 1 min, and remove the supernatant. Repeat this step three times. **Troubleshooting:** If there is a loss of beads while removing the supernatant, use an empty spin column, which was used for step 3.4.
6. Add 100 µL of 150 µg/mL DYKDDDDK peptide solution in PBS into a tube of step 6, and mix by rotator at 4 °C for 1 h.
7. Centrifuge the beads at 5000× *g* for 1 min and transfer the supernatant into a new tube. Store the recovered supernatant at 4 °C.
8. Prepare the His-binding column by packing 50 µL of His-beads into empty spin column followed by applying 5 CV of PBS.
9. Apply the sample from Step 7 to the column, close the cap of the column, and allow it to bind to the beads for 1 h at 25 °C by gently stirring it with a rotator.
10. Pass through the column by gravity flow and wash the beads by applying 5 CV of His-washing buffer to the column. Drain the buffer. Repeat this step three times.
11. Stop the flow and add apply 1.5 CV of His-elution buffer. Agitate the resin by gently stirring it for 1 h at 25 °C. Start the flow and collect the elution.
12. Exchange the buffer to PBS using a 3 k MWCO Centricon centrifugal ultrafilter. Add the eluted sample in the top part of the filter. Centrifuge at 10,000 rpm until the volume of sample is 0.1 mL.
13. Loading 0.5 mL of PBS and centrifuge at 10,000 rpm until the 0.1 mL of sample are remaining. Repeat this step three times.
14. Recover the buffer exchanged protein from the membrane, use a 200 µL pipette tip, and insert the tip in the bottom of the filter unit.
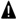
**CRITICAL STEP** Failure to wash the unreacted free dye may result in high background fluorescence.
15. Check the labeling efficiency and purity with 10 µL of sample by running an SDS-PAGE followed by scanning the gel using a fluorescence scanner.
16. Dilute the dialyzed protein to 1 mg/mL using PBS with 15% glycerol. Prepare aliquots of the samples, freeze them on dry ice, and lyophilize. Store the samples at −80 °C.

### 3.6. Fluorescence Measurements (Time for Completion: 1 Day)

1. To evaluate the quenching capacity, mix 2 nM Q-body and 250 µL of PBST or denaturant (7 M GdnHCl and 100 mM DTT) in quartz microcuvette.
2. Measure the emission intensity with excitation at 546 nm using an FP-8500 spectrofluorometer.
3. To measure the antigen-dependent fluorescence response of Q-body, mix 2 nM Q-body and 250 µL of PBST in a cuvette, and add various concentrations of 3-[(2S)-2-(methylamino)propyl]phenol, phenethylamine, or methoxyphenamine in 2 µL of PBST for titration to give final concentrations of 0 to 10^4^ µg/mL. As a control, add the same volume of PBST to normalize the signal.
4. Measure the emission intensity with excitation at 546 nm using an FP-8500 spectrofluorometer.
5. Draw fluorescence titration curves at the emission maxima of each spectrum using KaleidaGraph 4.5 (Synergy Software, Reading, PA, USA).

## 4. Results and Discussion

**Figure 2:**
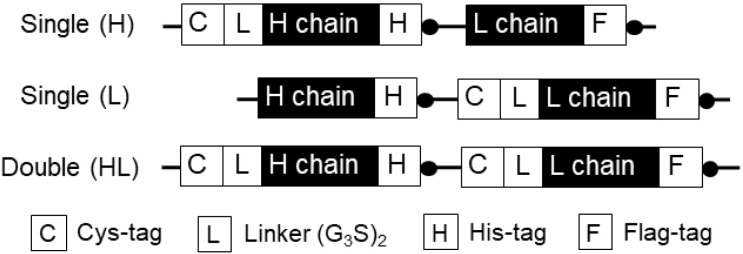
Schematic representations of Q-body expression genes with different fluorescent dye-binding sites.

The anti-MA scFv gene M9 used in this study was originally cloned and affinity-matured by G. Georgiou’s group [17]. Since this anti-MA antibody had the same number of Trp residues in both the heavy chain variable (VH) and light chain variable (VL) domains (three Trp residues in each domain), we constructed three different DNA genes with different sites for fluorophore-labeling: close to the H chain, to the L chain, or to both the chains. We inserted a Cys-tag for conjugating a dye at the N-terminal regions of the H and L chains (Figure 2). The anti-MA Fab gene with a single Cys-tag at the N-terminal region of the H/L chain was cloned into the pUQ1H/pUQ1L vector. Similarly, we generated a gene for a double-dye-labeled Q-body with two Cys-tags at the N-terminal regions of both the H and L chains. 

**Figure 3:**
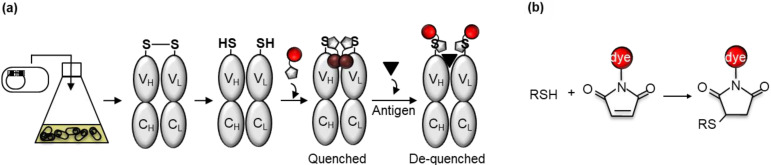
(**a**) Scheme for the construction of Fab type Q-body from *Escherichia coli*. Q-body with both H- and L-chain labeling sites is represented. Fab is expressed in *E. coli* cytoplasm and purified by IMAC via His-tag. After mild reduction of the exposed SH group of N-terminal Cys-tag, the dyes are conjugated and unbound dyes are eliminated; (**b**) schematic representation of maleimide-thiol reaction-based conjugating method for labeling a Fab with a fluorescent dye.

Previously, the VH and VL genes of clone M9 had been codon-optimized and subcloned into the pROX vector to generate a cell-free transcription-translation system-based anti-MA Q-body with a fluorophore located at the N-terminal region of the H chain [pROX1H(MeM9)] [9]. In this study, the DNA was subcloned into pUQ1H, pUQ1L, and pUQ2 to express each Fab in the cytoplasm of *E. coli* [8]. The anti-MA Fabs were expressed in soluble form and then purified by immobilized metal affinity chromatography (IMAC) via the His-tags. After a mild reduction of the cysteine thiol group of the Cys-tag, a maleimide-conjugated fluorescent dye was conjugated to the reduced thiol group utilizing the maleimide-thiol reaction (Figure 3). Next, the Q-bodies were tandemly purified by IMAC and anti-Flag affinity beads to remove the excess free dyes. The Q-bodies were then used for fluorescence measurements in the presence of denaturant or antigen.

**Figure 4:**
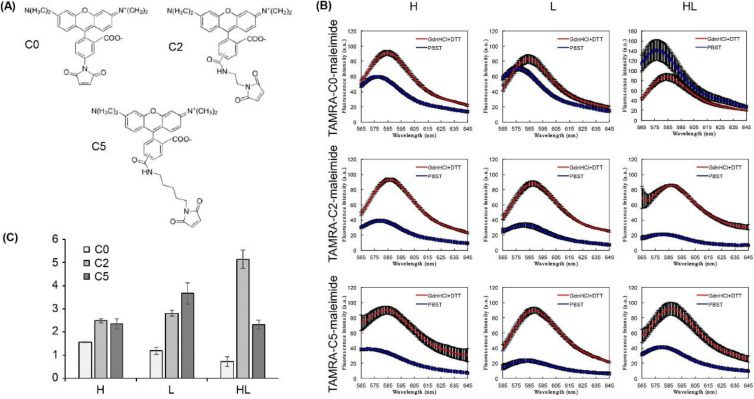
(**A**) Chemical structures of TAMRA-C0-maleimide, TAMRA-C2-maleimide, and TAMRA-C5-maleimide; (**B**) fluorescence intensities of Q-bodies in the presence of denaturants (7 M guanidine hydrochloride and 100 mM dithiothreitol) or PBST. Error bars represent ±1 SD (n = 3); (**C**) normalized fluorescence intensities of each Q-body, which were calculated by dividing the maximum fluorescence intensity in the presence of denaturants by the maximum in the presence of PBST. Error bars represent ±1 SD (n = 3).

Our previous study utilizing a Fab-type Q-body against bone Gla protein (BGP) showed that the differences in linker length between the maleimide and Cys-tag affected the fluorescent response of the Q-body in the presence of antigen [8]. Based on these results, we varied the spacer length between the dye and maleimide: TAMRA-CO-maleimide (C0), TAMRA-C2-maleimide (C2), or TAMRA-C5-maleimide (C5) (Figure 4a). We evaluated the maximum degree of quenching (quenching capacity) of each Q-body by measuring the fluorescence intensities in the presence of a denaturant (7 M guanidine hydrochloride and 100 mM dithiothreitol) and normalizing the values against those obtained under the non-denatured condition (PBST added instead of the denaturant). The responses of the denatured Q-bodies were 1.56 ± 0.03-, 1.19 ± 0.15-, 0.73 ± 0.38-, 2.48 ± 0.26-, 2.80 ± 0.24-, 5.13 ± 0.79-, 2.35 ± 0.36-, 3.67 ± 0.68-, and 2.31 ± 0.33-fold for the C0-labeled H, C0-labeled L, C0-labeled HL, C2-labeled H, C2-labeled L, C2-labeled HL, C5-labeled H, C5-labeled L, and C5-labeled HL, respectively (Figure 4b,c). For the C2-linker, the responses of two single dye-labeled Q-bodies (H and L) were similar, and these values were approximately two times lower than for the double dye-labeled Q-body (HL), as estimated based on the same number of Trp residues in the VH and VL domains. The C0/C5-linker Q-bodies did not show this trend. Although the three-dimensional structures of these Q-bodies have not been determined, we can state that the optimal position of dyes around the Trp residues affects the degree of quenching. We estimated that the C0 linker was too short to stay close to the Trp residues in VH as well as in VL. However, the C2 and C5 linkers were a suitable length to increase the quenching capacity.

**Figure 5:**
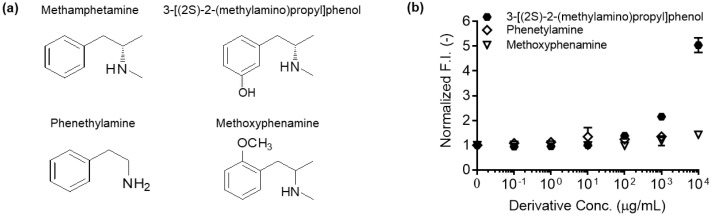
(**a**) Chemical structures of methamphetamine, 3-[(2S)-2-(methylamino)propyl]phenol, phenethylamine, and methoxyphenamine; (**b**) normalized fluorescence intensities of double TAMRA-C2-maleimide-labeled Q-bodies in the presence of 3-[(2S)-2-(methylamino)propyl]phenol, phenethylamine, or methoxyphenamine at the indicated concentrations. Methamphetamine itself was not used for generating the titration curve because illicit drug was not available for use in our laboratory. The normalized fluorescence intensity of each sample based on the fluorescence intensity at zero-dose was plotted. Error bars represent ±1 SD (n = 3).

The double TAMRA-C2-maleimide labeled Q-body had the highest quenching capacity and was used for testing the antigen dose-dependency. Since MA is an illicit drug, it was not generally available. Therefore, we used 3-[(2S)-2-(methylamino)propyl]phenol as a surrogate since the only structural difference was the addition of a hydroxyl group on the benzene ring (Figure 5a). The fluorescence of the double TAMRA C2-labeled Q-body was measured at different concentrations (0.1 to 10^4^ µg/mL) of 3-[(2S)-2-(methylamino)propyl]phenol. The fluorescence intensity increased to 4.40 ± 0.12- fold at 10^4^ µg/mL of 3-[(2S)-2-(methylamino)propyl]phenol, similar to the fluorescence intensity in the presence of denaturant (Figure 4c and Figure 5b). This indicated that the dye was almost completely de-quenched by the antigen. This data suggested that the fluorescence dye was successfully incorporated into the antigen-binding domain of the Fab fragment and displaced by antigen, and that quenching was removed in an antigen-concentration-dependent manner.

In our previous study, a single labeled Fab-type Q-body was generated through a cell-free translation and transcription system. The resultant Ultra Q-body showed MA-dependent fluorescence increases up to 7.2-fold [8]. The lower response of the Q-bodies generated in this study may reflect the differences in the dye conjugation method. The previously published cell-free based Q-body was generated with a TAMRA-tRNA conjugated to the amber-tag with the (GlySer)_2_ linker on the N-terminus of the H chain. Therefore, the distance between the dye and Trp was not the same as that of the TAMRA-C2-maleimide-labeled Q-body. As a primary reason for the higher EC_50_ of this double-labeled Q-body than the value of the single-labeled Q-body [8], we think that the dye-dye stacking occurred between the two dyes, resulting in the need of high concentration of antigen for de-quenching [10,11,12]. Additionally, we removed the cysteine residues at the C-terminal of the Fd and L chains, which are used for the disulfide bond between them, to increase the efficiency of site-specific labeling at the cysteine of the Cys-tag. This could have caused the Q-body to have somewhat lower antigen affinity and resultant lower sensitivity. Although the fluorescence response and sensitivity were lower than the cell-free based one, the *E. coli*-based approach for constructing Q-bodies have several advantages. First, the yield of Q-body produced by *E. coli* is higher than the cell-free system. Better yield increases cost efficiency, which is one of the major obstacles for the large-scale production of Q-bodies for practical applications. The expression of recombinant antibody fragments in *E. coli* has a higher yield than hybridoma-based production of full-sized antibodies, and such an inexpensive bacterial production may result in lower product costs. Thus, this concept holds promise as a general strategy for the development of a commercially available biosensor. Second, the maleimide-conjugated dye is less expensive to synthesize than the tRNA-conjugated dye, and has a wider range of colors. The wider range of multi-color labels for Q-bodies provides greater flexibility in developing multi-plex drug screening kits. Finally, to test the cross-reactivity of anti-MA Q-body against other MA derivatives, we used phenethylamine and methoxyphenamine. These chemicals have fewer structural similarities to those of MA than 3-[(2S)-2-(methylamino)propyl]phenol. The anti-MA Q-body exhibited a negligible response in the presence of phenethylamine or methoxyphenamine. The lack of response indicates that there is no cross-reactivity to other MA derivatives, which suggests that the anti-MA Q-body has a high MA selectivity (Figure 5).

We produced an *E. coli*-based Q-body against a hydroxyl MA derivative with the goal of developing a fast and convenient on-site MA screening kit. In particular, we focused on the number and position of fluorescent dyes with different linker lengths to produce a Q-body with an optimized response range. In addition, the Q-body reported here is highly selective to 3-[(2S)-2-(methylamino)propyl]phenol. The major advantage of the Q-body is that the test takes a few minutes of incubation followed by the fluorescence measurement. It is a single step test with no need for multiple incubation and washing steps, like those required for other conventional immunoassays such as ELISA. Therefore, this anti-MA Q-body has potential as a reagent in a drugs-of-abuse analysis platform when used in conjunction with a portable fluorophotometer. This type of rapid and simple assay tool may be useful in a range of areas, such as drug screening at the workplace, multiplexed point-of-care testing for simultaneous on-site detection of various stimulant drugs including cocaine, morphine, and suspicious substance identification, and monitoring patients during drug rehabilitation. Additionally, the Cys-tag-based Q-body-synthesis method reported in this study can be applied to the generation of Q-bodies of interest by fusing fluorescence dye to antibodies in a number of different antibody formats.

## Figures and Tables

**Table 1:** Sequence of template DNAs. (underline: tag for labeling or for non-labeling; bold: site for the same; italic: linker).

Template Name	Sequence
tgcHchain	atgtctaaacaaatcgaagtaaac**TGC**tctaatgagacc*ggtggcggttcaggcggcggatca*caggtccagctgcagcagtctggacctgagctggtgaagcctggggcttcagtgaaggtatcctgcaaggcttctggttacccatccactcgcttctacatctactgggtgaagcagagccacggaaagagccttgagtggattggaaatattgatccttacaatggtggtactacctacaaccagaagttcaagggcaaggccacattgactattgacaagtcctccaccacagcctacgtgcatctcaacagcctgacatctgaggactctgcagtctattactgtgcaggatttcattactccggccagttggataccgatgtctggggcgcagggaccaaggtcaccgtttcctcggccaaaacgacacccccatctgtctatccactggcccctggatctgctgcccaaactaactccatggtgaccctgggatgcctggtcaagggctatttccctgagccagtgacagtgacctggaactctggatccctgtccagcggtgtgcacaccttcccagctgtcctgcagtctgacctctacactctgagcagctcagtgactgtcccctccagcacctggcccagcgagaccgtcacctgcaacgttgcccacccggccagcagcaccaaggtggacaagaaaattgtgcccagggattgtggggggggttctgactacaaggacgacgatgacaagtaataa
tttHchain	atgtctaaacaaatcgaagtaaac**TTT**tctaatgagacc*ggtggcggttcaggcggcggatca*caggtccagctgcagcagtctggacctgagctggtgaagcctggggcttcagtgaaggtatcctgcaaggcttctggttacccatccactcgcttctacatctactgggtgaagcagagccacggaaagagccttgagtggattggaaatattgatccttacaatggtggtactacctacaaccagaagttcaagggcaaggccacattgactattgacaagtcctccaccacagcctacgtgcatctcaacagcctgacatctgaggactctgcagtctattactgtgcaggatttcattactccggccagttggataccgatgtctggggcgcagggaccaaggtcaccgtttcctcggccaaaacgacacccccatctgtctatccactggcccctggatctgctgcccaaactaactccatggtgaccctgggatgcctggtcaagggctatttccctgagccagtgacagtgacctggaactctggatccctgtccagcggtgtgcacaccttcccagctgtcctgcagtctgacctctacactctgagcagctcagtgactgtcccctccagcacctggcccagcgagaccgtcacctgcaacgttgcccacccggccagcagcaccaaggtggacaagaaaattgtgcccagggattgtggggggggttctgactacaaggacgacgatgacaagtaataa
tgcLchain	atgtctaaacaaatcgaagtaaac**TGC**tctaatgagacc*ggtggcggttcaggcggcggatca*gatattgttatgacccagtctccagcattcatgtctgcatctcctggggagaaggtcaccttgacctgcagtgccagctcaagtgtaagttccaccttcttgtactggtaccagcagaagccaggatcctcccccaaactctggatttatagcacatccaacctggcttctggagtcccttctcgcttcagtggcagtgggtctgggacctcttactctctcacaatcaacagcatggaggctgaagatgctgcctcttatttctgccatcagtggagtaattacccattcacgttcggaagtgggaccaagctggaaatcaaacgtgctgatgctgcaccaactgtatccatcttcccaccatccagtgagcagttaacatctggaggtgcctcagtcgtgtgcttcttgaacaacttctaccccaaagacatcaatgtcaagtggaagattgatggcagtgaacgacaaaatggcgtcctgaacagttggactgatcaggacagcaaagacagcacctacagcatgagcagcaccctcacgttgaccaaggacgagtatgaacgacataacagctatacctgtgaggccactcacaagacatcaacttcacccattgtcaagagcttcaacaggaatgagtgtggggggggttctcatcatcatcatcatcattaa
tttLchain	atgtctaaacaaatcgaagtaaac**TTT**tctaatgagacc*ggtggcggttcaggcggcggatca*gatattgttatgacccagtctccagcattcatgtctgcatctcctggggagaaggtcaccttgacctgcagtgccagctcaagtgtaagttccaccttcttgtactggtaccagcagaagccaggatcctcccccaaactctggatttatagcacatccaacctggcttctggagtcccttctcgcttcagtggcagtgggtctgggacctcttactctctcacaatcaacagcatggaggctgaagatgctgcctcttatttctgccatcagtggagtaattacccattcacgttcggaagtgggaccaagctggaaatcaaacgtgctgatgctgcaccaactgtatccatcttcccaccatccagtgagcagttaacatctggaggtgcctcagtcgtgtgcttcttgaacaacttctaccccaaagacatcaatgtcaagtggaagattgatggcagtgaacgacaaaatggcgtcctgaacagttggactgatcaggacagcaaagacagcacctacagcatgagcagcaccctcacgttgaccaaggacgagtatgaacgacataacagctatacctgtgaggccactcacaagacatcaacttcacccattgtcaagagcttcaacaggaatgagtgtggggggggttctcatcatcatcatcatcattaa
